# Ultraviolet-germicidal-irradiation-based reduction of airborne bacteria in pulmonary function test rooms: a quality improvement report

**DOI:** 10.1016/j.infpip.2026.100571

**Published:** 2026-08-05

**Authors:** Yo-Han Seo

**Affiliations:** Department of Laboratory Medicine, Gwangju Veterans Hospital, Gwangju, Republic of Korea

**Keywords:** Air microbiology, Hospital environment, Infection control, Pulmonary function test, Ultraviolet rays

## Abstract

**Background:**

Pulmonary function test (PFT) rooms involve frequent patient turnover and can generate respiratory aerosols during forced expiratory manoeuvres. This quality improvement (QI) project evaluated observed changes in culturable airborne bacteria (CAB, cfu/m^3^) under routine clinical operation when ultraviolet germicidal irradiation (UVGI)-related engineering controls were applied singly or in combination, to inform pragmatic infection-prevention options for PFT rooms.

**Methods:**

CAB was sampled by impaction at two fixed locations in the PFT room using a consistent protocol with repeated measurements. The baseline/control condition was defined as routine operation with supplemental devices OFF (ceiling UV-C OFF and portable air-circulation UV sterilizer OFF) under the specified heating, ventilation, and air conditioning (HVAC) setting, and intervention conditions were assessed using the same protocol. Lobby measurements were used only as contextual building-environment comparators.

**Results:**

The combined operation of ceiling UV-C, the portable air-circulation UV sterilizer, and HVAC yielded the lowest observed CAB, while ceiling UV-C alone produced the largest reduction among single interventions in this setting. Representative isolates included common skin/environmental taxa (e.g., *Staphylococcus hominis*), consistent with human occupancy and resuspension.

**Conclusions:**

In this real-world QI evaluation, UV-C-containing interventions were associated with lower culturable airborne bacterial counts in the PFT room, with the lowest observed values under combined engineering controls. These findings should be interpreted as culture-based room-level observations under routine workflow conditions, not as evidence of complete air disinfection or respiratory pathogen reduction.

## Introduction

Pulmonary function testing (PFT) rooms are environments with a high risk of cross-infection by airborne micro-organisms due to the frequent visits of patients with respiratory diseases and the aerosols and droplets generated during testing. Additionally, the 2016 Pulmonary Function Test Guidelines state that “for diseases with a high possibility of droplet transmission (e.g., tuberculosis), environmental control, such as ventilation, air filtration, or ultraviolet irradiation of the air, is necessary” [1]. The 2025 Evaluation Indicators for Special Health Examination Institutions inquire about the installation of ventilation systems with external air discharge structures or disinfection facilities, such as ultraviolet sterilizers that can be used continuously during work [2].

The 2019 revised guidelines for pneumoconiosis health diagnosis and PFT/assessment quality control restrict the range of acceptable ventilation systems to windows, negative pressure generators, and independent ventilation systems (ventilation systems connected to the overall heating, ventilating, and air conditioning system (HVAC) system are not recognized) [3]. The infection control criteria also specify that “UV sterilizers must be products that are proven to reduce airborne bacteria by 80% or more in accordance with SPS-KOUVA AS 01-1889:2022 standards or equivalent domestic and international standards, and must cover an area larger than the examination room, with disinfection power maintained during the examination” [4]. To comply with these guidelines and evaluation criteria, PFT rooms must be located on the outer walls of buildings with windows or be equipped with independent ventilation systems, along with ultraviolet (UV) sterilizers that can be operated continuously during testing.

UV light is a form of electromagnetic radiation with wavelengths ranging from approximately 100 to 400 nm, and it is classified into UV-A, UV-B, UV-C, and vacuum UV based on wavelength. UV-A, with wavelengths between 315 and 400 nm, is primarily used for tanning and photocatalytic activation. UV-B, with wavelengths between 280 and 315 nm, is used in dermatological treatments and meteorological equipment. UV-C, with wavelengths between 200 and 280 nm, is used for the disinfection of surfaces, air, and water. Vacuum UV, with wavelengths between 100 and 200 nm, is used to remove trace organic materials in semiconductor processes [5].

A Korean consumer safety survey reported that some marketed UV sterilization products did not deliver the advertised UV-C performance or used light sources outside the claimed germicidal range [5]. Therefore, the light-source type, wavelength, irradiance, and validated operating conditions should be confirmed rather than inferred from product labeling alone. Devices often referred to as 'lamps' use either high-voltage-discharge (HVD) lamps or light-emitting-diode (LED) technology, which can have different germicidal performance characteristics. Commercial validation of UV-C-based air-cleaning devices is commonly performed under controlled challenge conditions, such as enclosed-chamber aerosol tests using surrogate bacteriophages or filter-pathway challenge tests. These certification-style tests are useful for standardized performance evaluation; however, they may not directly represent room-scale performance in occupied clinical spaces, where airflow patterns, device placement, ventilation, and room mixing influence microbial transport and removal [6,7].

In this context, this study aimed to verify the reduction of airborne micro-organisms in PFT rooms using UVGI systems under real environmental conditions and to propose efficient infection control measures. PFT rooms are characterized by frequent patient entry and exit, as well as continuously running HVAC systems, which introduce various environmental factors. To address these challenges, this study investigates the effectiveness of UV sterilizers by examining the changes observed after using different types of sterilizers for varying durations under such conditions. Because PFT rooms are continuously influenced by patient/staff movement and ventilation operation, this quality improvement (QI) evaluation focuses on observed changes under routine conditions using a clearly defined baseline/control state and a consistent sampling protocol, rather than attempting to estimate device ‘efficacy’ under controlled chamber conditions.

## Methods

### Study design and operational context

This work was conducted as a QI project in an active hospital PFT room during routine operation. Sampling was performed during routine daytime operation of the PFT room. The room operates by scheduled appointments, with approximately 20–25 patients examined per day and approximately 2–4 tests/h during busier workflow periods. During routine workflow, each patient entered and left the room for testing, and one to two staff members were usually present. Door opening occurred intermittently with patient entry, exit, and staff movement. These occupancy-related factors were not experimentally controlled and were considered part of the real-world QI context.

### Baseline/control definition

The baseline/control condition was defined as routine operation with the supplemental devices OFF (ceiling UV-C OFF; portable air-circulation UV sterilizer OFF) under the specified HVAC setting used for baseline. Each intervention condition was assessed using the same sampling protocol and locations, and repeated measurements were obtained for each condition to evaluate reproducibility under routine variability.

**Figure 1:**
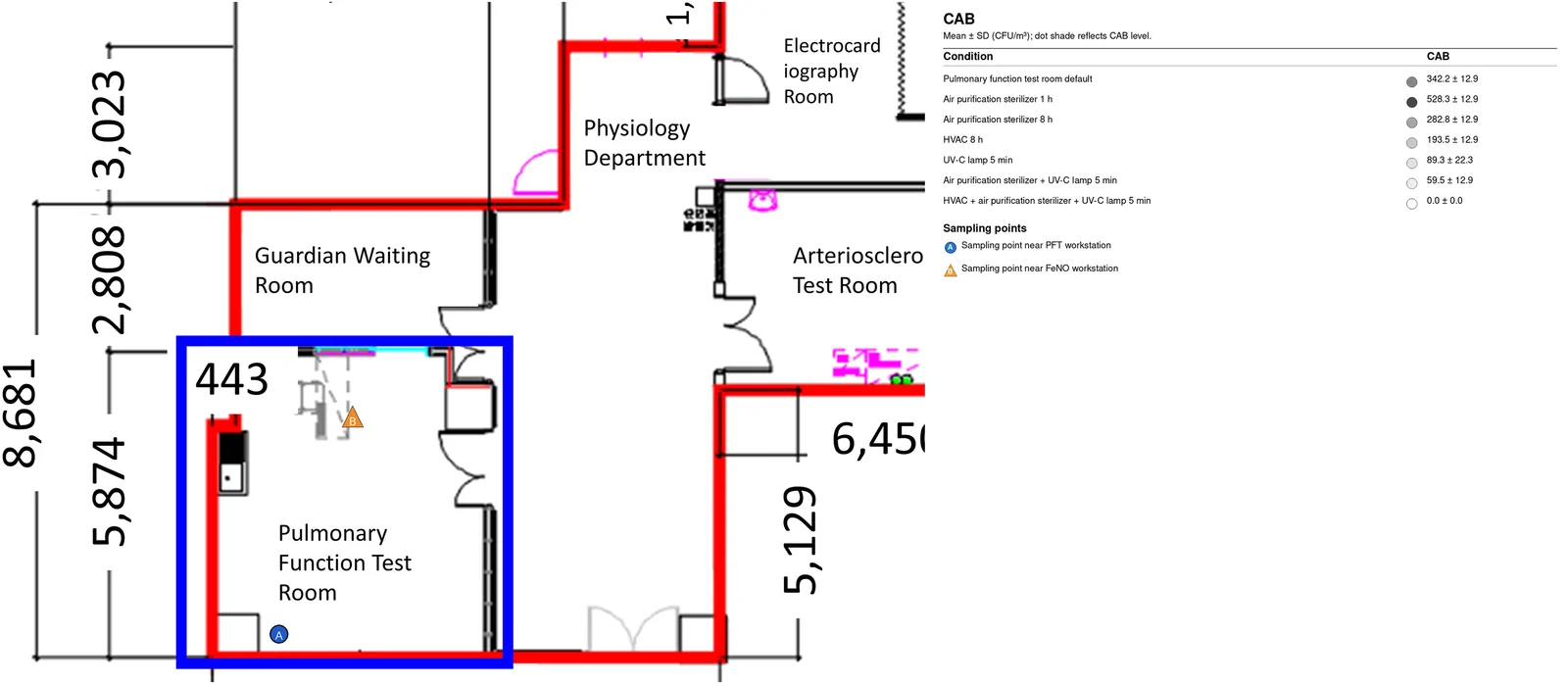
Schematic floor plan of the pulmonary function test room showing sampling points (A and B) and the culturable airborne bacteria levels by condition. FeNO, fractional exhaled nitric oxide; HVAC, heating, ventilation, and air conditioning; SD, standard deviation; CAB, culturable airborne bacteria; UV, ultraviolet.

This study was conducted in the PFT room (space volume: 57 m^3^) of a 500-bed general hospital located in Gwangju Metropolitan City (Figure 1). The UV lamps used in this study were cost-effective models priced around 50,000 KRW per unit, with the following specifications: lamp power 20 W, voltage 80 V, current 250 mA, output 9.5 W, and a UV irradiance of 85.0 μW/cm^2^, with a lifetime of 8000 h. It is important to note that the output wattage differs from the lamp power wattage and that even 20-W lamps from different manufacturers can have varying output wattage. Additionally, the remaining lifespan of the lamp, the material and angle of the reflector, and other physical factors must be considered when evaluating sterilization performance. For an accurate assessment of the effectiveness of air purification sterilizers, Bernoulli's principle and precise calculations for exposure time and installation position should also be taken into account.

**Figure 2:**
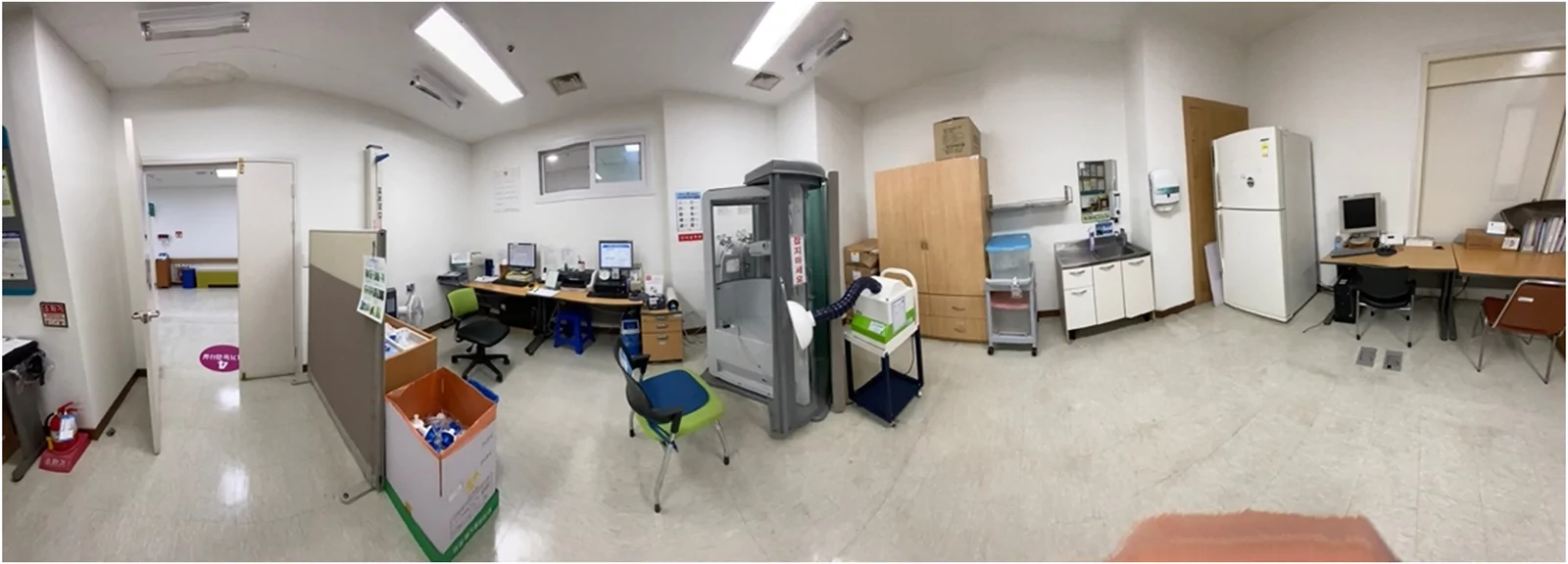
Panoramic view of the pulmonary function testing room.

This PFT room originally has a single entrance, one window installed in the upper section, and an HVAC system on the ceiling consisting of four supply vents and two exhaust vents. Additionally, four 20W UV-C lamps were newly installed on the ceiling (Figure 2). For safety, direct UV-C exposure to occupants was avoided by operating the UV-C irradiation as a brief post-test procedure when the room was unoccupied and by following local safety protocols. Radiometric irradiance mapping (mW/cm^2^) was not performed; therefore, delivered UV dose (mJ/cm^2^) could not be calculated. Fixture model/specifications and placement were reported to support reproducibility as an implementation-focused QI report.

**Figure 3:**
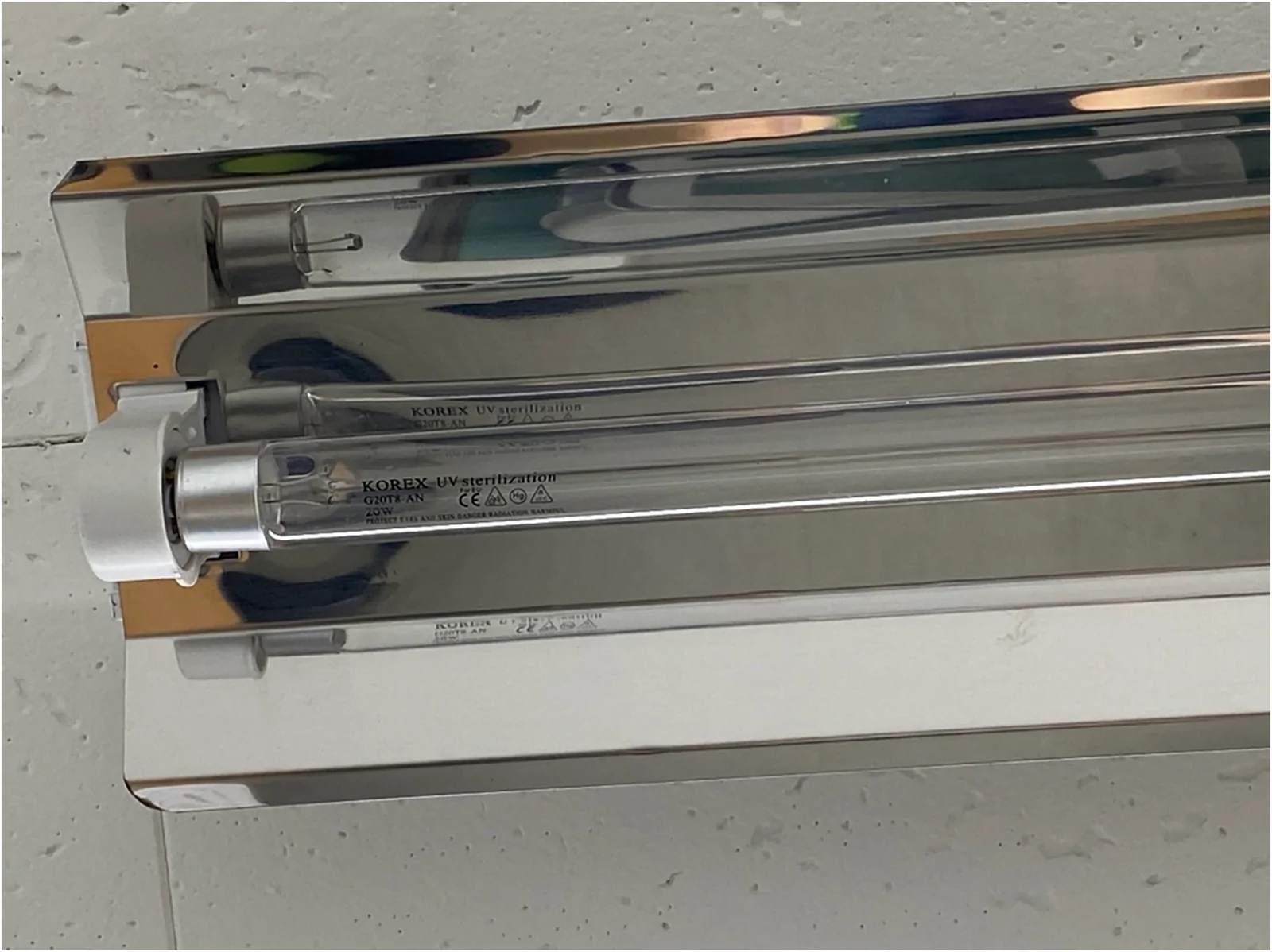
20-W UV-C Lamp installed on the ceiling.

**Figure 4:**
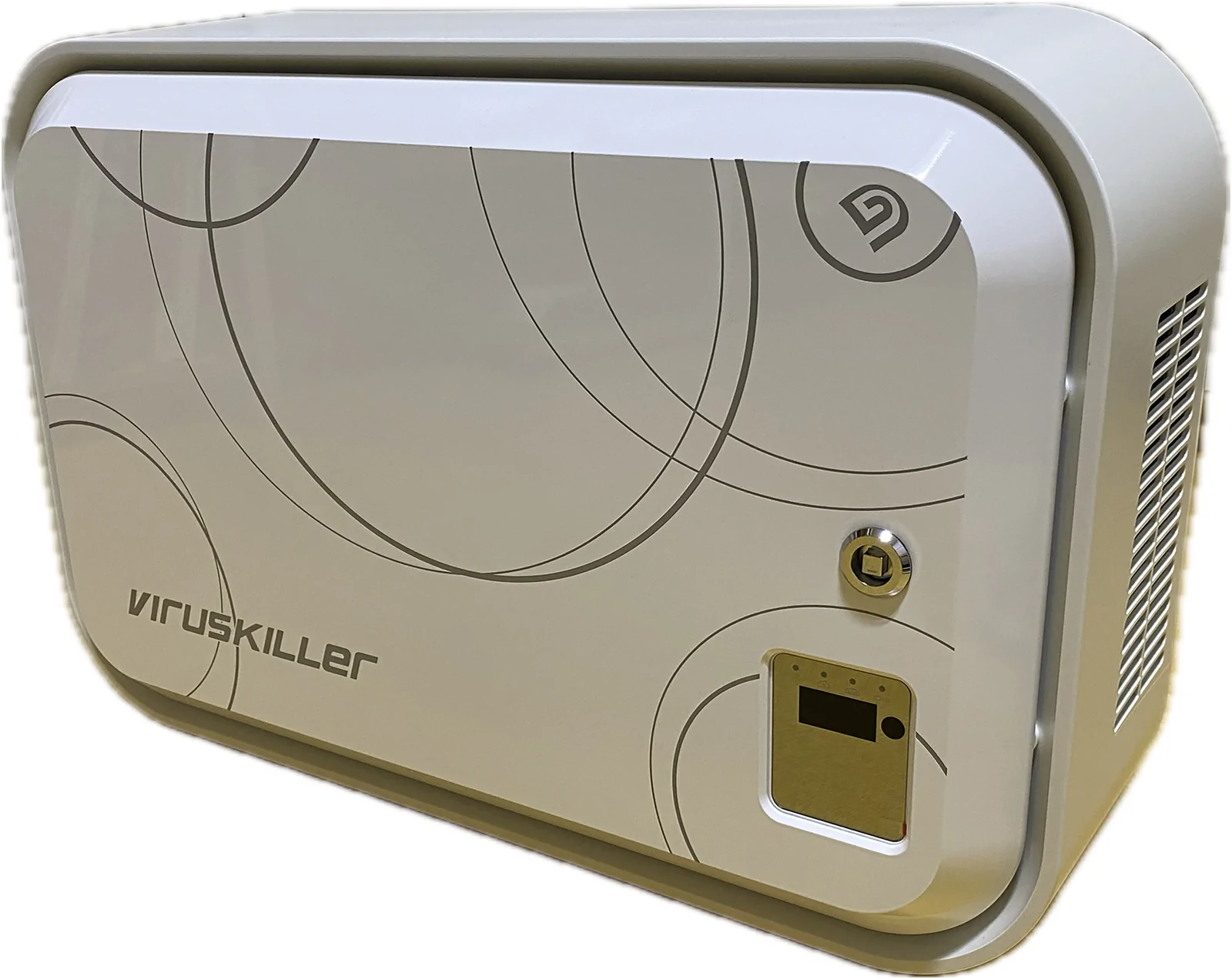
Air purification sterilizer.

The airborne micro-organism concentrations were compared across the hospital's PFT room, the waiting area of the testing room, and the hospital's main lobby, as well as the PFT room with the hospital's HVAC turned off. The comparison was made by using ceiling-mounted UV lamps (20 W, four units) (Figure 3), an air purification sterilizer (VK Clinic, INB Korea) (Figure 4), and an HVAC system (48,500 CMH, 1008 L/m), either individually or in combination. In practical terms, air purification sterilizer recirculates a fraction of room air through an internal disinfection pathway (UV/plasma/filters), and its real-room impact depends on airflow rate, placement, and mixing.

All air-quality measurements were performed with reference to the revised Indoor Air Quality Test Standard (National Institute of Environmental Research Notice No. 2023-1, issued 5 January 2023) [8].

Samples were collected at two points because the total floor area was less than 10,000 square meters. Air samples were taken near the spirometry equipment and exhaled nitric oxide measurement equipment at heights of 1.2–1.5 m from the floor. Culturable airborne bacteria were measured using an airborne bacteria sampler (KAS-110, KEMIK Corporation) via the impaction method. Air was collected for 2 min at a flow rate of 22.4 L/min, and the collected air was impacted on to a Tryptic Soy Agar (TSA) plate. The plates were incubated at 37°C for 48 h, after which the colony forming units (cfu) were counted and expressed as airborne concentration (cfu/m^3^). Each measurement was repeated three times, and the results were calculated as an average value. Air samples were collected at two fixed locations within the PFT room (FeNO-side and PFT-side). At each location, three replicate impaction samples were collected sequentially within the same sampling session using identical sampler settings (flow rate and sampling duration). The arithmetic mean of the three replicates was used as the representative value for each location under each operating condition.

The culturable airborne bacteria concentration (cfu) was calculated using the formula:

$$\text{CAB}\;\text{concentration}\;\left(\text{CFU}/m^{3}\right)=\left[\text{colony}\;\text{count}\;\left(\text{CFU}\right)/\left\{\text{sampling}\;\text{flow}\;\text{rate}\;\left(L/\min\right)\times \text{sampling}\;\text{time}\;\left(\min\right)\right\}\right]\times 1000$$

The reduction rate of airborne bacteria, based on the initial CFU concentration of the PFT room, was calculated using the formula:

ReductionRate(%)=((InitialConcentration−PostTreatmentConcentration)/InitialConcentration)×100

Isolated individual colonies were analysed by 16S rRNA sequencing, and nucleotide sequences were examined using the BLASTn Search program from the NCBI (National Center for Biotechnology Information) database. This analysis was outsourced to Cosmo Genetech (Daejeon, Korea). Identification was performed for representative culturable isolates only; viruses and non-culturable microbiota were not assessed in this QI evaluation.

In addition to collecting air samples, temperature and humidity were measured at heights of 1.2–1.5 m using an in/out thermohygrometer (Sanyo, Japan). PM10 and PM2.5 concentrations were measured using a real-time monitor (Radic Air Quality Sensor, INBair, Korea). Formaldehyde, volatile organic compounds (VOCs), and carbon dioxide concentrations were measured using an indoor air quality meter (nearBeBe Air, Nearbebe, Korea). PM2.5/PM10 monitoring was used as an ancillary environmental indicator and was not intended to distinguish droplet versus respirable aerosol fractions; aerodynamic particle size distribution was not measured in this QI evaluation.

The terms “main hospital lobby,” “pulmonary function test waiting area,” and “pulmonary function test room default” presented in the results represent the default air conditions in different locations. The “air purification sterilizer for 1 h” condition was set considering the typical hospital lunch break duration of approximately 1 h. Meanwhile, the “air purification sterilizer for 8 h” condition was included to evaluate the microbial reduction when the sterilizer operates continuously for a full 8-h work shift.

Regarding the UV-C lamp exposure time, it needed to be set below 15 min, as 15 min is the standard patient-to-patient ventilation time recommended in testing guidelines. Considering the irradiance level of the UV-C lamp used in this study, a 5-min exposure was determined, as this duration can achieve a 2-log reduction of most bacteria. This study was approved by the Institutional Review Board (IRB) of Gwangju Veterans Hospital (IRB approval no.: device 2023-12-1).

## Results

**Figure 5:**
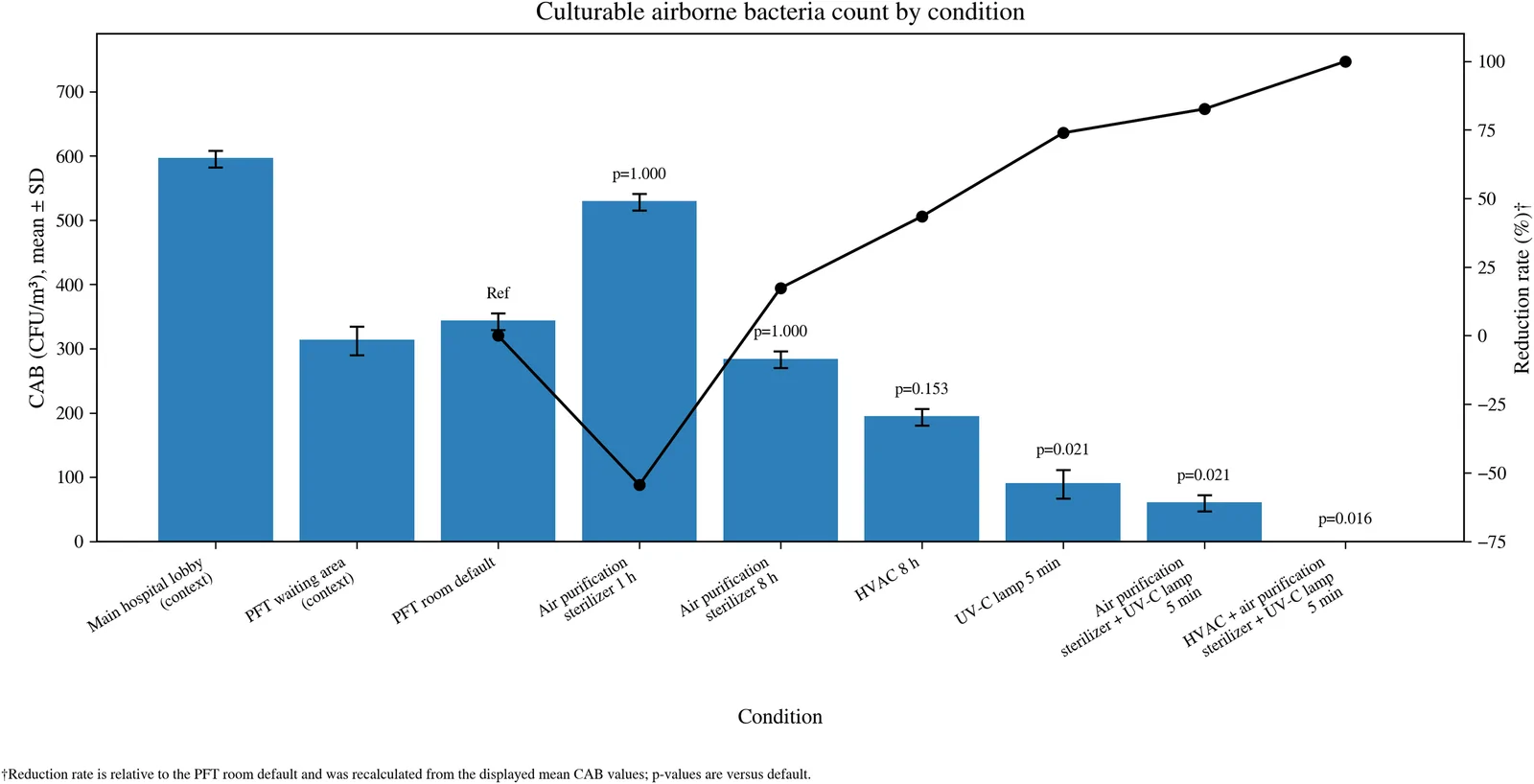
Culturable airborne bacteria (CAB) count by condition. CFU, colony-forming units; HVAC, heating, ventilation, and air conditioning; SD, standard deviation; UV, ultraviolet. Reduction rates are relative to the pulmonary function test room default; *P*-values are versus default.

Culturable airborne bacteria (CAB) counts in the PFT waiting area, main hospital lobby, and PFT room default condition were 312.5 ± 22.3, 595.3 ± 12.9, and 342.2 ± 12.9 cfu/m^3^, respectively, all below the reference value of 800 cfu/m^3^. Among the PFT-room intervention conditions, the air sterilizer-only 1-h condition showed a higher CAB count than the default condition, whereas UV-C-containing conditions showed lower CAB counts. CAB counts were 89.3 ± 22.3 cfu/m^3^ after UV-C lamp exposure alone and 59.5 ± 12.9 cfu/m^3^ after combined air sterilizer and UV-C exposure. No culturable bacteria were detected under the sampling conditions used for the combined HVAC + air sterilizer + UV-C condition. Detailed mean ± standard deviation values, reduction rates, and *P*-values are presented in Table I and Figure 5. For PFT-room conditions, six measurements per condition were used (two fixed points, A and B; triplicate at each point). Overall differences among conditions were tested using the Kruskal–Wallis test (H = 34.37, *P*=5.7 × 10^−6^), followed by post-hoc comparisons versus the default condition with Holm-adjusted *P*-values. UV-C–containing conditions showed statistically significant reductions compared with default.

**Table I:** Culturable airborne bacteria count by condition

Condition	CAB (cfu/m^3^)	Reduction rate (%)^a^	*P*^b^
Main hospital lobby (context)	595.3 ± 12.9 (*N* = 3)	–	–
Pulmonary function test waiting area (context)	312.5 ± 22.3 (*N* = 3)	–	–
Pulmonary function test room default	342.2 ± 12.9 (*N* = 6)	0.0	Ref
Air purification sterilizer 1 h	528.3 ± 12.9 (*N* = 6)	-54.4	1.000
Air purification sterilizer 8 h	282.8 ± 12.9 (*N* = 6)	17.4	1.000
HVAC 8 h	193.5 ± 12.9 (*N* = 6)	43.5	0.153
UV-C lamp 5 min	89.3 ± 22.3 (*N* = 6)	73.9	0.021
Air purification sterilizer & UV-C lamp 5 min	59.5 ± 12.9 (*N* = 6)	82.6	0.021
HVAC & air purification sterilizer & UV-C lamp 5 min	0.0 ± 0.0 (*N* = 6)	100.0	0.016

CAB, culturable airborne bacteria count; cfu, colony forming units; HVAC, heating, ventilation, and air conditioning; UV, ultraviolet. Data are presented as mean ± standard deviation.

a Reduction rate calculated relative to the PFT room default. Negative values indicate an increase vs default.

b Holm-adjusted P-values from post-hoc comparisons versus default after an overall Kruskal-Wallis test. Because the six measurements per condition were sequential within-session samples from two fixed locations in one room, these P-values should be interpreted as exploratory.

Most of the identified bacteria were *Staphylococcus hominis* subsp., *Staphylococcus pseudolugdunensis* strain B006, *Pantoea ananatis* strain 1846, *Bacillus oceanisediminis* strain H2, and *Micrococcus* sp. strain EB239, which are commonly found on the skin or in soil. The average temperature across all conditions was 24.7 ± 0.2°C, and the average humidity was 34.5 ± 2.4%. Carbon dioxide levels ranged from 477 to 666 ppm. Using the current Korean medical-facility reference limits of 0.08 mg/m^3^ for HCHO and 0.40 mg/m^3^ for TVOC, both limits were exceeded under the air purification sterilizer 1 h, HVAC 8 h, UV-C lamp 5 min, and air purification sterilizer + UV-C lamp 5 min conditions. Both HCHO and TVOC were within these limits under the default, air purification sterilizer 8 h, and combined HVAC + air purification sterilizer + UV-C lamp conditions. PM10 and PM2.5 concentrations ranged from 2 to 6 microg/m^3^ and were below the corresponding medical-facility limits of 75 and 35 microg/m^3^, respectively (Supplementary Table S2). These measurements were contextual indicators rather than primary study outcomes. CO_2_ was monitored as a contextual indicator of occupancy and ventilation rather than as a primary outcome variable. In the conditions in which CO_2_ was recorded, values ranged from 477 to 666 ppm, suggesting that sampling was conducted under generally acceptable background ventilation and occupancy conditions. No formal CO_2_-CAB correlation analysis was performed. Formaldehyde (HCHO) and total VOCs were measured as contextual indicators of indoor air quality rather than as primary outcomes. In the subset of conditions in which these variables were recorded, HCHO ranged from 0.019 to 0.700 mg/m^3^ and total VOCs ranged from 0.128 to 5.8 mg/m^3^. The lowest recorded concentrations were observed under the combined HVAC + air purification sterilizer + UV-C condition (Supplementary Table S2). This pattern may reflect enhanced air exchange/removal and temporal variation in indoor pollutant source strength, but the finding should be interpreted cautiously because these variables were not primary study endpoints.

## Discussion

The main finding of this QI evaluation was that UV-C-containing interventions were associated with lower CAB counts in the PFT room under routine workflow conditions. The lowest observed CAB count occurred under the combined HVAC + air sterilizer + UV-C condition, whereas the air-sterilizer-only condition did not show a consistent reduction. These findings should be interpreted as room-level observations under the specific sampling and workflow conditions used in this study. Representative culturable isolates included common skin/environmental taxa (e.g., *S. hominis*), consistent with human occupancy and resuspension.

The air purification sterilizer used in this study consumes 80 W and contains eight UV lamps. It includes 40 nanotubes coated with photocatalyst. The filter system consists of a prefilter and a HEPA filter, and the area of coverage is 66 square meters. The manufacturer reported a sterilization efficacy of 96.3% in its technical specification (data on file); this manufacturer-reported value was not independently verified in the present evaluation.

In all conditions, the airborne bacteria counts were already below the Indoor Air Quality Management Act (effective 15^th^ March 2024), which sets the standard for culturable airborne bacteria in medical facilities at less than 800 cfu/m^3^. This study aimed to describe observed changes in CAB counts under different engineering-control conditions in a real PFT room, rather than to determine device efficacy under controlled experimental conditions. Ozone generation is a potential safety concern for some UV-based devices, particularly at ozone-generating wavelengths [5]. Ozone was not quantitatively evaluated in this QI study; therefore, no conclusion regarding device-related ozone generation can be drawn from these data. Future implementation assessments should include direct indoor ozone monitoring when the lamp emission spectrum or device design presents this risk.

UV-C inactivates microorganisms primarily by inducing nucleic-acid photolesions, particularly cyclobutane pyrimidine dimers and (6-4) photoproducts between adjacent pyrimidines. These lesions interfere with DNA replication and transcription, thereby reducing microbial viability. Effectiveness depends on delivered UV dose, wavelength, distance, exposure time, medium, and the microorganism [9].

Conventional ventilation and sterilization systems still have limitations in preventing the transmission of pathogens through the air. Respiratory pathogens can be transmitted through droplets and aerosols; SARS-CoV-2, the virus that causes COVID-19, is one example. The relative contribution of larger droplets, finer aerosols, and contaminated surfaces varies by pathogen and setting [[10], [11], [12], [13]].

Air intake-based sterilization devices have several limitations in removing pathogens from the air. These devices typically draw in air through an intake, filter or irradiate it with UV light, and then release it. However, heavy droplets and aerosols often do not enter the intake, or the air intake speed is insufficient to effectively remove them. As a result, these devices mainly treat smaller airborne particles, while larger droplets and bacteria remain, continuing to pose infection risks.

In contrast, ceiling-mounted UV-C sterilization systems have the advantage of more effectively treating airborne pathogens. UV-C light, which has a short wavelength, destroys the DNA of micro-organisms, leading to their sterilization. UV-C lamps installed on the ceiling can directly irradiate airborne pathogens, thus preventing their transmission. If air is adequately mixed, ceiling-mounted UV-C can irradiate airborne microorganisms during controlled, unoccupied-room cycles; the present evaluation did not assess continuous operation during occupancy.

Research on air sterilization systems using plasma generators has shown that superoxide anions can modify oxygen molecules in the air, demonstrating powerful sterilization effects [14]. However, such systems still depend on airflow to distribute ions throughout the indoor air, limiting their effectiveness if the air does not circulate well. Conversely, ceiling-mounted UV-C systems are less affected by airflow and can sterilize large areas of air in a relatively short time.

Air intake-based sterilization devices may show strong performance under controlled chamber or filter-pathway test conditions, but their real-room impact can be affected by airflow patterns, placement, ventilation, and mixing. In this study, the air sterilizer-only condition did not produce the lowest CAB counts, whereas UV-C-containing conditions were associated with lower culture-based bacterial counts. However, these findings should be interpreted only as changes in culturable airborne bacteria under the sampling conditions used, not as evidence of respiratory pathogen reduction or transmission prevention. The focus of this study was to compare observed CAB changes under feasible engineering-control conditions in a real PFT room, rather than to rank devices by intrinsic disinfection efficacy. The decision to install a total of 80 W (four 20-W lamps) on the ceiling was made to align with the UV lamps in air purification sterilizers, which typically use 10-W UV lamps consuming 80 W in total. The primary objective was to ensure a comparable wattage rather than to calculate the precise UV dose required for the given space.

However, such an approach would turn this into a controlled experimental study, deviating from the primary aim of this research, which is to propose practical airborne micro-organism reduction strategies for PFT rooms. Unlike manufacturers' tests conducted under highly controlled conditions to verify microbial elimination efficacy, this study focuses on real-world application strategies. When installing UV lamps, it is recommended to consult with UV specialists and carefully assess the specific spatial conditions to ensure optimal performance.

This study has some limitations. First, the space was difficult to control due to the intermittent movement of patients requiring PFTs. However, this was seen as a helpful variable in conducting a more practical evaluation of the system's effectiveness. Second, variables such as the presence of reflective metal plates, the sterilization efficacy depending on the distance of the UV-C lamp [15], the fact that it is an air sterilization method rather than a surface sterilization method, and the use of four separate UV-C lamps rather than just one made it difficult to calculate the exact sterilization time for the specific space. A 5-min UV-C exposure was associated with a lower CAB count under the sampled condition. Because only one UV-C exposure duration was evaluated and delivered UV dose was not measured, the minimum effective exposure time cannot be determined from this study. Future evaluations should compare multiple exposure durations with irradiance or dose measurements [16,17]. Third, UV-induced DNA damage in bacteria can be partially reversed through repair mechanisms, including photoreactivation under visible light exposure and dark repair, which may reduce apparent UV disinfection efficacy if organisms are subsequently exposed to light. In addition, UV susceptibility varies across bacterial species and physiological states, which may contribute to heterogeneous reductions in culturable counts across conditions. These factors may introduce residual variability in real-world settings and warrant cautious generalization of the observed reductions [[18], [19], [20]]. Fourth, all patients were scheduled appointments, and patient throughput during the study period was known at the room level. The patient population was predominantly older male veterans. However, this QI study was designed to evaluate room-level engineering controls rather than patient-level determinants of airborne bacterial burden. Accordingly, individual factors such as age, sex, occupational exposure, clothing contamination, and rural or agricultural background were not systematically analysed and may have contributed to day-to-day variability. Future prospective studies should incorporate patient-level variables together with environmental monitoring.

We did not measure aerosol particle size distributions (e.g., APS/OPC), and therefore cannot attribute observed CAB changes to specific aerosol size fractions generated during/after spirometry. This study used CAB as a pragmatic indicator; therefore, non-culturable bacteria, fungi, and respiratory viruses were not captured. Ventilation dilution is a major potential confounder. Because ACH was not directly measured for each condition, we cannot apportion the observed CAB reductions to UVGI versus increased air exchange/clean-air delivery; the 0 cfu/m^3^ result should be interpreted as ‘no culturable bacteria detected’ under the sampling volume, culture conditions, and workflow conditions used in this study, rather than as complete absence of airborne micro-organisms.

The combined HVAC + air sterilizer + UV-C condition showed the lowest observed CAB count under the sampling conditions used. PFT rooms in special health examination institutions should be equipped with air purification sterilizers that can be used continuously during work, but combining them with UV-C lamps is expected to produce even greater reductions. For PFT rooms that do not yet have such systems, installing UV-C lamps and irradiating the air for a short time after PFTs could result in significant airborne micro-organism reduction. Lack of irradiance mapping limits transferability of the findings to rooms with different geometries, reflectance, and air-mixing conditions.

From an implementation perspective, feasibility depends on capital cost (equipment and installation), operating cost (electricity), and maintenance. Operating cost can be estimated as (power rating, W/1000) × operating hours × local electricity tariff, and maintenance includes periodic lamp/filter replacement and performance checks. Ceiling-mounted direct UV-C may be a relatively cost-efficient option for controlled, unoccupied-room disinfection when appropriate safety interlocks and operating procedures are used; this study did not evaluate continuous operation during occupancy, whereas air intake-based sterilizers may show strong chamber-test performance but can be constrained by airflow mixing, placement, and filter/UV module maintenance in real-world rooms.

## CRediT authorship contribution statement

**Yo-Han Seo:** Writing – review & editing, Writing – original draft, Visualization, Validation, Supervision, Software, Resources, Project administration, Methodology, Investigation, Funding acquisition, Formal analysis, Data curation, Conceptualization.

## Data availability

All data generated or analysed during this study are included in this published article. Additional datasets are available from the corresponding author upon reasonable request.

## Ethics approval and consent to participate

This study was approved by the Institutional Review Board of the Gwangju Veterans Hospital (IRB No. device 2023-12-1) and performed in accordance with the principles of the Declaration of Helsinki.

## Funding sources

This study was supported by a VHS Medical Center Research Grant (VHSMC 23050), Republic of Korea.

## Conflict of interest statement

The author declares no conflicts of interest.
