# Supplementary material for: Ultraviolet-germicidal-irradiation-based reduction of airborne bacteria in pulmonary function test rooms: a quality improvement report
Source: Infect Prev Pract. 2026 Aug 5;8(4):100571. doi: 10.1016/j.infpip.2026.100571 (PMC13573740; doi:10.1016/j.infpip.2026.100571)
Supplement: Supplementary file 1 [file mmc1.docx]

**Supplementary Table S2. Environmental parameters measured under each experimental condition.**

| **Condition** | **HCHO (mg/m³)** | **TVOC (mg/m³)** | **PM2.5 (µg/m³)** | **PM10 (µg/m³)** | **CO₂ (ppm)** |
| --- | --- | --- | --- | --- | --- |
| Pulmonary function test room default | 0.062 | 0.392 | 2 | 2 | 563 |
| Air purification sterilizer 1 hour | 0.089 | 0.428 | 6 | 6 | 518 |
| Air purification sterilizer 8 hours | 0.032 | 0.128 | 3 | 4 | 477 |
| HVAC 8 hours | 0.700 | 5.8 | 6 | 6 | 666 |
| UV-C lamp 5 minutes | 0.191 | 0.755 | 4 | 4 | 611 |
| Air purification sterilizer & UV-C lamp 5 minutes | 0.162 | 0.728 | 2 | 2 | 636 |
| HVAC & air purification sterilizer & UV-C lamp 5 minutes | 0.019 | 0.197 | 2 | 2 | 632 |

Abbreviations: HCHO, formaldehyde; TVOC, total volatile organic compounds.
